# A neurotechnological aid for semi-autonomous suction in robotic-assisted surgery

**DOI:** 10.1038/s41598-022-08063-w

**Published:** 2022-03-16

**Authors:** Juan Antonio Barragan, Jing Yang, Denny Yu, Juan P. Wachs

**Affiliations:** 1 School of Industrial Engineering, Purdue University, West Lafayette, IN USA; 2 School of Medicine (Adjunct), Indiana University, Indianapolis, IN USA

**Keywords:** Cognitive control, Biomedical engineering

## Abstract

Adoption of robotic-assisted surgery has steadily increased as it improves the surgeon’s dexterity and visualization. Despite these advantages, the success of a robotic procedure is highly dependent on the availability of a proficient surgical assistant that can collaborate with the surgeon. With the introduction of novel medical devices, the surgeon has taken over some of the surgical assistant’s tasks to increase their independence. This, however, has also resulted in surgeons experiencing higher levels of cognitive demands that can lead to reduced performance. In this work, we proposed a neurotechnology-based semi-autonomous assistant to release the main surgeon of the additional cognitive demands of a critical support task: blood suction. To create a more synergistic collaboration between the surgeon and the robotic assistant, a real-time cognitive workload assessment system based on EEG signals and eye-tracking was introduced. A computational experiment demonstrates that cognitive workload can be effectively detected with an 80% accuracy. Then, we show how the surgical performance can be improved by using the neurotechnological autonomous assistant as a close feedback loop to prevent states of high cognitive demands. Our findings highlight the potential of utilizing real-time cognitive workload assessments to improve the collaboration between an autonomous algorithm and the surgeon.

## Introduction

In minimally invasive surgery, the introduction of robotic platforms has improved the surgeon dexterity and visualization, but it has not reduced the number of surgical staff in the Operating Room (OR)^1^. As the leading surgeon sits in a console separated from the patient, he depends on surgical assistants to perform support tasks on the patient side. These support tasks include exchanging the robotic tools, handling sutures and specimens, and providing suction and irrigation^2^. Among the assistants’ responsibilities, blood suction and irrigation tasks are critical to maintain a clear surgical field and avoid complications during a procedure often resulting from bleeding^3^.

Effective use of the suction and irrigation tool is a critical skill for a successful Robotic Minimally Invasive Surgery (RMIS). In particular, blood suction can facilitate proper hemorrhage control by allowing the surgeon to localize the source of bleeding and treat it in a timely manner. Effective bleeding control requires the surgeon and the surgical assistant to coordinate their actions during the procedure^4^. When hemorrhages events in minimally invasive procedures are not properly controlled, the surgeon must undock the robot and switch to open surgery^5^, which can further increase the risk of postoperative complications such as morbidity, infections, and subsequent surgeries^6^.

To increase the surgeon’s independence, teleoperable flexible suction tools, such as the ROSI (Remotely Operated Suction Irrigation System), have been developed^7,8^. These novel devices can be held with the robot’s grasper, allowing the surgeon to directly control the suction tool from the console. Paradoxically, having the leading surgeon in charge of blood suction leads to additional cognitive demands (since there are other tasks that the surgeon is responsible for) that can result in delays and, potentially, medical errors^7,9^. These additional cognitive demands can be particularly detrimental for the less experienced surgeons as they diverge their attention from the main procedure^10^.

To alleviate these needs, we propose utilizing NOCAAS, a NeurOtechnology based Cognitive Aware Autonomous System, to provide assistance during hemorrhage control situations. Such a framework would help to improve performance and reduce the cognitive demands of the medical staff. This system leverages recent Artificial Intelligence (AI) advancements to segment automatically the endoscopic images and then extract navigational cues necessary for effective suction of blood accumulations. To benchmark our system performance, a surgical simulator was developed. This simulator was used to generate mock bleeding events while the user performed a running suture exercise^11^. In this regard, the autonomous system worked concurrently with the user to provide suction on the surgical workspace.

To enhance the human–robot collaboration, the autonomous agent adapts its behavior according to the surgeon’s mental state and needs at a precise moment^12^. In this regard, a cognitive workload detection algorithm based on electroencephalogram (EEG) and eye tracking sensors was designed and implemented to improve the robot awareness of the surgeon’s mental state. This framework allowed us to estimate the cognitive demands of the user in real-time and adapt the autonomous assistant accordingly. Such a neurotechnology based system has the potential to reduce the surgeon’s dependence on the surgical assistant by making the robot more responsive to the surgeons’ needs. In the cases where the surgeon is performing the suction by himself, our system would prevent the prolonged states of high cognitive load that can deteriorate their performance and response to unexpected situations^13,14^.

Two user studies with a first-generation Da Vinci surgical robot validated our approach. This robot was controlled by using the open-source hardware and software of the Da Vinci Research Kit (DVRK)^15^. The first study aimed to validate the performance of our cognitive workload detection system. The second study aimed to demonstrate that working with NOCAAS can result in better performance and lower cognitive demands on the user. To achieve this, we evaluated the user performance in a surgical exercise under two modalities: manually controlled-suction tool (*manual* modality) and autonomy controlled-suction tool (*autonomous* modality). In the former, the user changed between the teleoperation of the suction tool and the main instrument arms. In the latter, the robotic assistant provided automatic blood suction directly. In both modalities, performance and workload metrics were collected to assess the effect of the AI system on task performance and cognitive demands.

## Results

Results for this work are divided into two different sections. First, a computational experiment was performed to assess the performance of deep learning models to predict mental states from a set of physiological markers. In the second experiment, the developed neurotechnology based cognitive workload prediction models were used to trigger the assistance of an autonomous suction assistant. The goal of this experiment was to demonstrate that an autonomous system can be more effective in its intervention by obtaining real-time information about the user’s mental state in RMIS.

To assess the performance of our cognitive workload assessment system, two datasets of physiological signals were collected from users performing different training surgical exercises with the DVRK robot. In each dataset, the difficulty of the task was increased to elicit mental states of high cognitive workload in the users. In this regard, physiological signals were labeled as *low cognitive load condition* (LCL) or *high cognitive load condition* (HCL). The first dataset included only EEG data from 8 subjects that performed a peg transfer task under two teleoperation conditions: normal (LCL) and inverted (HCL). For the second dataset, EEG and eye tracker signals were collected from 10 subjects that performed a needle pass exercise on a custom-made bleeding simulator. The task performed with no bleeding events was assumed as (LCL) and the bleeding condition as (HCL). These categories were chosen as it has been well documented that bleeding complications are a significant surgical stressor that affects technical and non-technical skills in less experienced surgeons^10^. Results from dataset 1 showed that EEG-only cognitive workload models required longer input signals of at least 80 s to achieve the best results. To reduce the sequence length of the signals, eye tracking signals were added to dataset 2. This led to accurate prediction models with sequences as short as 25 s long. In terms of prediction models for dataset 1, the best prediction accuracies were obtained by using a recurrent architecture based on LSTM cells. For dataset 2, as temporal information was not as critical, a feed-forward neural network was adopted which is computationally more efficient than the LSTM model.

To assess the effects of NOCAAS in task performance, a user study was conducted with 10 participants. In this experiment, the users performed a surgical exercise under two modalities: *manual controlled-suction tool* condition (MS) and *autonomy controlled-suction tool* (AS). In each condition, objective performance and subjective workload metrics were collected to demonstrate the benefits of our autonomous robotic assistant. The objective performance metrics were based on motion statistics (see Methods section) of the robot’s Patient-Side-Manipulators (PSM).The included supplementary video shows the user and the autonomous system working together to perform the simulated task. The DVRK robot has three independent manipulators on the patient side (PSMs) which can be controlled via the surgical console; however, only two of the PSMs can be controlled simultaneously by the user. In the MS condition the user swapped between the control of the main instruments arms (PSM1 and PSM2) and the suction tool control (PSM3) by tapping a pedal in the console. In the AS condition, the suction tool (PSM3) was controlled by our autonomous system. Every time the user swapped the control from the main instrument arms to the suction tool was counted as a tool switching event.

### Experiment 1: evaluation of the neurotechnology based real-time workload assessment system

#### Classification accuracy

**Figure 1:**
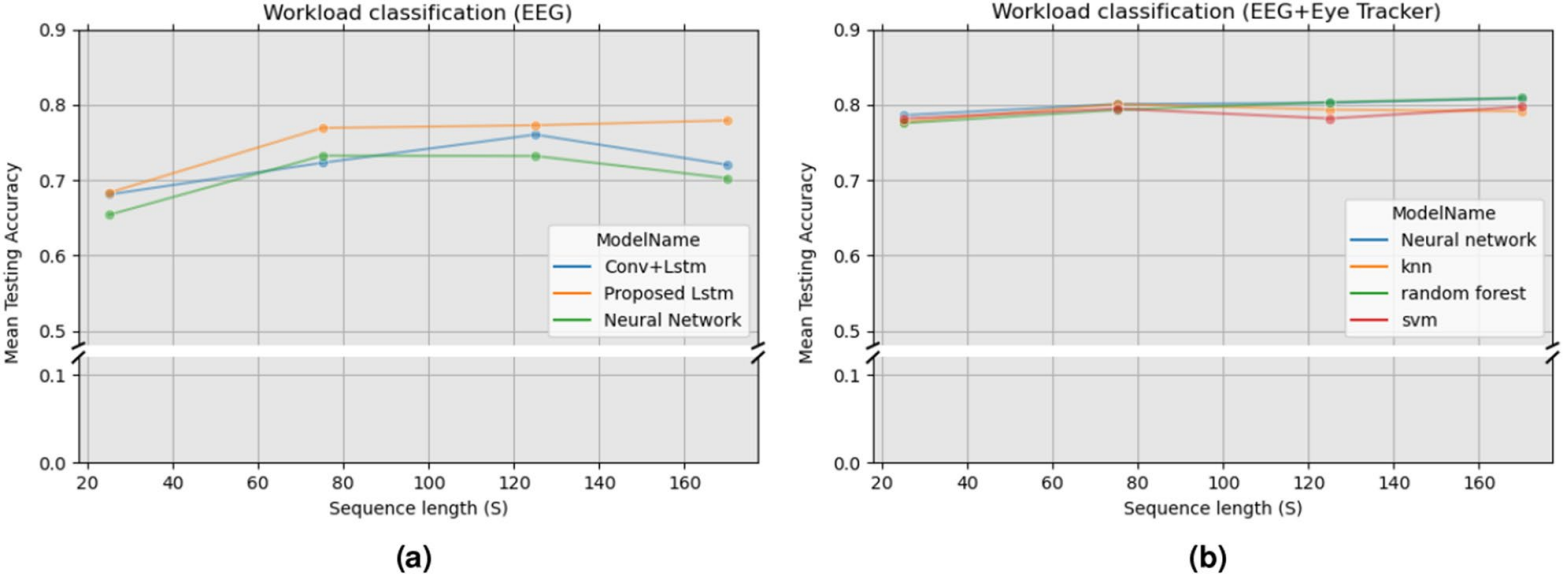
(**a**) Correlation between accuracy and sequence length in dataset 1. The best performing model is our proposed recurrent architecture based on LSTM cells. (**b**) Correlation between accuracy and sequence length for dataset 2. The best performing was a feed-forward neural network.

Figure 1a,b show the prediction accuracies of proposed models for dataset 1 and dataset 2 respectively. Additionally, the accuracy was calculated for multiple sequence lengths of physiological signals. For workload classification in dataset 1 (EEG-only), we used a recurrent LSTM architecture (referred as to “proposed LSTM“). This model was tested against the following baseline models: a convolutional LSTM model^16^ and a feedforward neural network. For dataset 2 (EEG-Eye tracker), we proposed using a Neural Network model. This model was tested against the following baseline models: KNN, random forest, SVM.

Results from dataset 1 can be seen in Fig. 1a. Here it can be seen a positive correlation between the sequence length and the classification accuracy. Increasing the sequence length from 10 to 100 s improved the model’s prediction accuracy from 68% to 78%. Using EEG spectral features from 5 s windows, a recurrent model based on bidirectional LSTM cells was proposed to predict the user’s mental state. Compared to the convolutional LSTM and the neural network, the proposed model obtained the highest prediction accuracies at every sequence length (see Table 1a). The best classification accuracy of our model was 78% at a sequence length of 170 s. These results indicate that EEG signals are strong predictors of the user’s mental state.

Results from dataset 2 can be seen in Fig. 1b. In this scenario, it was observed that the mean prediction accuracy is not as correlated with the input sequence length as in dataset 1. Results from the proposed model for this dataset (neural network) showed a 2% increment in the accuracy by increasing the sequence length from 25 to 75 s. After, 75 s improvements in prediction accuracy are less than 1%. Compared to the tested traditional machine learning models, our classifier obtains better performance for every sequence length except 170 s. The best classification accuracy of our proposed model is 80.25% with a sequence length of 125 s. Table 1 summarizes all the results from the computational experiment.

**Table 1:**
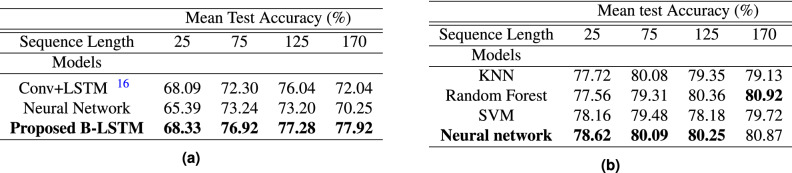
Accuracy analysis for multiple models and sequence lengths. (a) Accuracy analysis for dataset 1. (b) Accuracy analysis for dataset 2.

Highest accuracy values are given in bold.

#### Physiological analysis of EEG features

**Figure 2:**
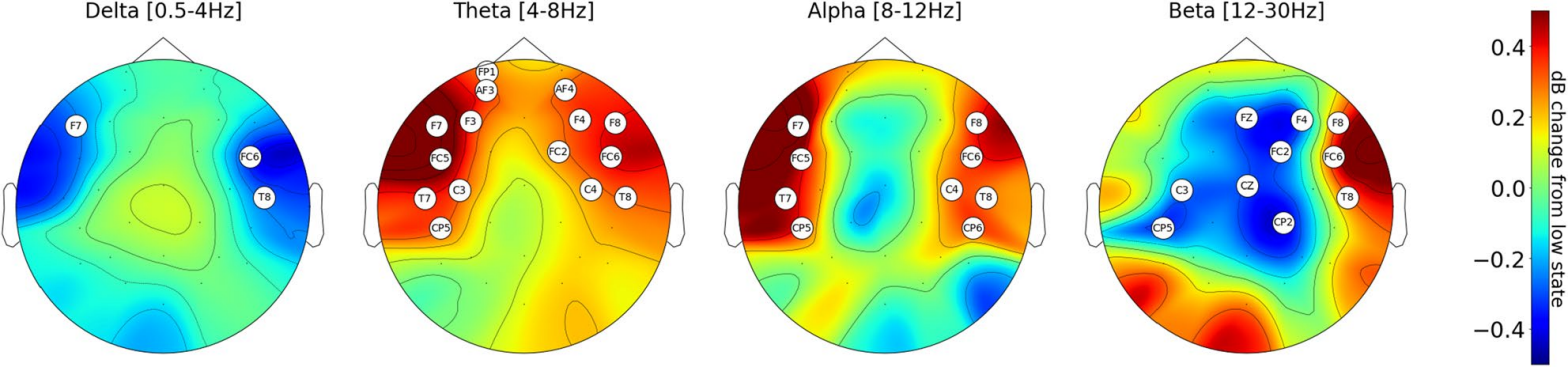
Mean scalp topography plot on the condition of high cognitive load for all the users with baseline subtraction. The baseline for all the channels was calculated with the data from the low cognitive state. Red areas represent increased oscillation activity in the condition of high cognitive workload while blue areas represent inhibition of the spectral activity.

Figure 2 shows the resulting scalp topographical maps of the EEG spectral features from dataset 1. In these plots, only channels having a difference of more than 0.2dB between the HCL and LCL conditions were shown. It was observed suppression of Delta activity on channels F7, FC5, and T8 in the HCL condition. In other words, the delta activity in the aforementioned channels was found to be lower in HCL condition compared to the LCL. Theta activity on channels FP1, AF3, AF4, F7, F3, F4, F8, FC5, FC2, FC6, T7, C3, C4, T8, CP5 and alpha activity on channels F7, F8, FC5, FC6, T7, C4, T8, CP5, CP6 were found to be higher in HCL than in LCL condition. Finally, Beta activity increased on F8, FC6, T8, OZ while it was suppressed on FZ, F4, FC2, C3, CZ, CP5, and CP2 on HCL. Overall these results indicate that most of the channels presenting significant differences due to cognitive load are in the frontal and temporal lobes of the brain. Moreover, the state of high cognitive workload was mainly characterized by an increase of theta and alpha activity.

### Discussions

Results from our computation experiment indicate that EEG spectral features allowed for a 78% prediction accuracy of the user’s mental workload when using 170 s of data. The amount of data required at the input of the model can be reduced to 25 s by including eye tracker features. Taking into account that the OR is a fast-changing environment, reducing the amount of data required for robust mental workload estimation while maintaining the robustness of the prediction could be a critical requirement for clinical application of this technology. Lastly, physiological analysis of the EEG spectral features of dataset 1, indicated an increase in the theta and alpha oscillations of the EEG signals in the fronto-temporal channels.

### Experiment 2: integration of robotic autonomous assistant and cognitive prediction module

Objective metrics results are presented in Table 2. The mean and standard deviation were calculated for each metric to compare between the autonomous (*AS*) and manual suction modalities (*MS*). Completion time under the *AS* ($334 \pm 141$) was 152 s lower compared to the *MS* modality ($486 \pm 210$). These differences were found statistically significant. In terms of collaboration fluency, *AS* enabled concurrent activity 23% of the time. Additionally, *AS* reduced the PSM1 and PSM2 idle time respectively by 27% and 18%. There were no statistical differences in the autonomous assistant (PSM3) idle time. These results imply an increment of the users’ attention on the main task in the *AS*. The velocity of the main instruments (PSM1 and PSM2) was found significantly higher in the *AS* modality. The respective velocity increments of PSM1 and PSM2 were 0.26cm/s and 0.17cm/s. The PSM3 velocity was not statistically different in both conditions. Finally, no statistically significant differences were found in the percentage of the detected blood accumulations. In the AS condition, the users had the option to take the control back of the suction tool if required. Nevertheless, very few users utilized this option as seen by the tool changing event metric in the AS condition.

Cognitive metrics are given in Table 3. These metrics were divided between the subjective metrics from the NASA-TLX^17^ questionnaires and objective metrics from the cognitive workload sensing framework. Similarly, mean and standard deviation were calculated to compare between experimental conditions. All components of NASA-TLX were found significantly lower in the *AS* modality. Additionally, the overall workload score in *AS* ($23 \pm 12$) was reduced 15 points compared to *MS* ($35 \pm 11$). Both these results indicate, the user experiencing lower cognitive demands when working with the autonomous system. The average cognitive index was not found statistically different between the two conditions.

### Discussions

Results from experiment 2 indicate the suction assistant allowed faster completion of the surgical task and reduced the perceived cognitive demands compared to the manual teleoperation of the suction tool. These results highlight that secondary tasks in RMIS such as blood suction can result in considerably higher mental demands for the teleoperator of the surgical robot. In this regard, the introduction of autonomous technologies into the OR can improve surgical care by allowing the surgeon to focus on the main steps of the procedure.

## General discussions

In this work, we introduce the design and implementation of an autonomous robotic assistant for the task of blood suction in RMIS. Our system used a combination of computer vision architectures to automatically navigate the surgical field to provide assistance to the surgeon who teleoperated the main instrument arms of the surgical robot. Additionally, a real-time cognitive workload sensing system was developed to provide the robotic assistant with information on the user’s mental state. This information was used to trigger the suction events, in moments of high mental demands for the user rather than using visual triggers from endoscopic images. The proposed neurotechnology based workload estimation system was based on EEG and eye tracker signals and obtained a prediction accuracy of 80% percent. Overall, results indicate that our proposed autonomous algorithms for the blood suction subtask can lead to better performance and lower mental demands.

**Table 2:** Objective performance metrics results. Statistically significant results ($\alpha =0.05$) were highlighted in bold.

Type	Name	Mean (std), N=10		T-test	
		Autonomy	Manual	T-statistic	p-value
Time	Clutching time (s)	2.96 (6.51)	14.84 (11.33)	− 3.211	**p** < **0.1**
	completion time (s)	334.11 (141.61)	486.19 (210.36)	− 4.480	**p** < **0.01**
Collaboration	Concurrent activity (%)	0.23 (0.07)	0 (0)	9.907	**p** < **0.01**
	Psm1 idle time (%)	0.21 (0.08)	0.48 (0.09)	− 9.139	**p** < **0.001**
	Psm2 idle time (%)	0.37 (0.15)	0.55 (0.13)	− 9.014	**p** < **0.001**
	Psm3 idle time (%)	0.7 (0.08)	0.82 (0.05)	− 3.047	0.014
Motion	Psm1 velocity (cm/s)	0.98 (0.24)	0.72 (0.2)	4.776	**p** < **0.001**
	Psm2 velocity (cm/s)	0.73 (0.17)	0.56 (0.19)	6.853	**p** < **0.001**
	Psm3 velocity (cm/s)	0.65 (0.17)	0.37 (0.09)	3.977	**p** < **0.01**
Events	Tool changing events	0.8 (1.23)	11.2 (8.57)	− 4.221	**p** < **0.01**
	Clutching events	0 (0)	3.7 (2.75)	− 4.254	**p** < **0.01**
Blood	Percentage blood (%)	0.14 (0.04)	0.13 (0.05)	0.593	0.568

**Table 3:** Nasa-TLX results and measured cognitive workload. Statistically significant results ($\alpha =0.05$) were highlighted in bold. The NASA-TLX is a 10-point Likert scale questionnaire that divides the workload demands into 6 components: effort, frustration, mental demand, performance, physical demand, and temporal demand.

Type	Component	Mean (std), N=10		T-test	
		Autonomy	Manual	t-statistic	p-value
Nasa-TLX	Effort	4.25 (2.81)	6.05 (2.44)	− 5.125	**p** < **0.001**
	Frustration	3.40 (2.09)	5.95 (2.09)	− 5.517	**p** < **0.001**
	Mental demand	4.30 (2.75)	6.45 (2.20)	− 5.018	**p** < **0.001**
	Performance	2.65 (2.27)	3.90 (2.61)	− 3.926	**p** < **0.01**
	Physical Demand	4.55 (2.66)	6.15 (3.10)	− 3.320	**p** < **0.01**
	Temporal Demand	3.85 (2.33)	6.60 (1.96)	− 6.942	**p** < **0.001**
	WorkloadScore	23.00 (12.41)	35.10 (11.56)	− 6.181	**p** < **0.001**
Workload prediction	Cognitive Index	0.527 (0.254)	0.585 (0.230)	− 1.666	0.14

### Real-time cognitive workload measurements

Real-time cognitive load assessment from physiological signals can improve performance and the learning of surgical skills in RMIS. Nevertheless, many challenges still need to be addressed, such as the reduced generalization of the prediction models due to the high variability of physiological signals. In this work, we collected two datasets of EEG and eye tracker signals in the context of RMIS. In these datasets, different mental demands were elicited by increasing the task difficulty. Using this data, we developed two deep learning models for the problem of classifying physiological signals into states of *low cognitive load* (LCL) and *high cognitive load* (HCL).

For the first dataset, only EEG signals were collected. As a prediction model, we proposed a recurrent neural network based on LSTM cells. This model proved suitable for predicting cognitive workload, as EEG signals can be converted into a sequence bandpower spectral coefficients that have an appropriate structure for a recurrent model. The prediction accuracy of the models was evaluated as the sequence of spectral coefficients increased. Additionally, we used a session-to-session evaluation scheme where the training and testing data belonged from different sessions. This evaluation allowed us to determine our models’ robustness to the day-to-day variability of physiological signals.

In this scenario, the proposed models obtained a mean classification accuracy of 79.2% when using EEG segments of over 100 s. Thus implying that the sequence length of spectral coefficients and the accuracy of the models’ predictions are positively correlated. In other words, there exists a trade-off between the inference speed and the accuracy of the models, since longer sequences of coefficients would result in prediction delays. In this regard, the sequence length of the recurrent models can be fine-tuned according to the application. If accurate predictions are required, then the sequence length should be increased at the cost of increasing the model’s latency. However, in real-time settings, the sequence length can be decreased to reduce prediction delays.

For the second dataset, a combination of EEG features and eye tracker features was collected. In this scenario, we proposed a feed-forward prediction model, as the eye tracker features did not depend on temporal information. Our proposed models achieved an accuracy of $80\%$ with a sequence length of over 100 s. The main benefit of introducing eye tracker features into the classification of cognitive workload is that the sequence length can be reduced to 25 s without compromising the prediction accuracy. Compared to the other baseline models (KNN, Random Forest, and SVM), the proposed neural network obtained the best prediction accuracy. Overall, results indicate that combining eye tracker and EEG features improves the robustness of the workload prediction system in RAS.

To better understand the driving features of the classification models, we created scalp plots from the EEG signals of dataset 1. This graphical representation allowed us to localize which channels presented the biggest differences between the states of LCL and HCL. Additionally, it allows us to identify specific cognitive functions that played a role during the experiment based on cognitive load theory^18^. First, Delta activity has been linked to the theory of Attention networks proposed by Corbetta et al.^19^. This theory states the existence of two different and competing attention mechanisms, one dedicated to processing external sensory information and another dedicated to internal concentration. Delta activity has been shown to be more active when internal concentration is required and suppressed on tasks where sensory information is needed^20–22^. For our experiment, a suppression of Delta activity could imply that the users were highly dependent on the visual feedback during the inverted teleoperation condition.

Theta band activity has been associated with increasing use of working memory capacity^23^. Additionally, Puma et al. reported increased theta and alpha activity levels when increasing the number of subtasks in a multitask testing environment^24^. These studies align with the increasing levels of theta and alpha activity found in the fronto-temporal channels during the high workload conditions. Overall, these results indicate the effectiveness of spectral band power features to discriminate between states of high and low cognitive workload.

Physiological sensing technology will become more common in the OR daily activities, as these sensors are integrated into commercial systems. In the case of robotic surgery, integrating physiological sensors in the surgical console will enable real-time measurements of physiological signals with very small disruptions on the surgeons’ workflow. In this regard, our work serves as significant preparative steps to enable robotic assistance to mitigate the effects of cognitive load in surgical performance.

### A neurOtechnology based cognitive aware autonomous system (NOCAAS)

For the second experiment, the cognitive workload prediction module was used to provide real-time information of the user’s mental state to the suction robotic assistant. In other words, the robot actions would only be activated when the system detected a state of High Cognitive Load. In this regard, the autonomous assistance, NOCAAS, can be viewed as a feedback mechanism to alleviate the surgeon’s cognitive demands during a procedure. A user study comparing the cognitive triggered autonomous system against manual teleoperation of the suction tool was conducted. Objective surgical performance metrics and cognitive metrics were calculated to validate the benefits of our system. In terms of surgical performance, the cognitive autonomy allowed the user to complete the suturing exercise 162 s faster than manual teleoperation. This result could be attributed to the improvements in the collaboration fluency between the robot and the user. On the autonomous modality, the idle time of the main instruments was reduced by 32%. This result can also be interpreted as having the user work 32% more time on the main procedure when collaborating with the autonomous system. Additionally, it is highlighted that the suction tool was active the same percentage of time during both conditions (PSM3 idle time showed no statistically significant differences). This result suggests that the motions of the suction tool were more efficient when the autonomous algorithm oversaw the teleoperation.

In terms of cognitive demands, NASA-TLX scores indicate users felt the surgical exercise was less demanding while working with autonomy. These results have two important implications. First, it indicates the importance of allowing novice users to fully concentrate on the main surgical exercise to improve surgical performance. Second, it highlights the effectiveness of our system in handling bleeding events. On the other hand, the cognitive index showed no statistically significant differences between the experimental conditions. This finding could be explained by additional challenges created by the autonomous system, such as the occlusion of the surgical view. Certain positions inside the cavity resulted in unavoidable occlusions from the suction tool. This most likely resulted in some additional mental demands that were not present in the manual teleoperation. This problem can be alleviated by providing autonomy with information on the current surgical step performed by the user. In this regard, the autonomy can plan suctions in locations where occlusion is unavoidable at better timings.

Overall, the presented solution is a good compromise between full autonomy and complete teleoperation. Although our system cannot distinguish the root cause of the cognitive load increase, i.e., whether is bleeding or other surgical concerns, experimental results demonstrate that the provided assistance improves task performance when triggered by high cognitive loads events.

## Methods

In this section, it is described the design and development of both the proposed cognitive workload sensing module and the autonomous robotic assistant. Additionally, the protocols to collect the physiological signals and the user study to test the autonomous system performance are described.

### Neurotechnology based cognitive load assessment module

#### Physiological sensors and synchronization

EEG recordings were made with a 32 channel G.Nautilus with active electrodes (Gel-based) from G.tec medical engineering GmbH, Austria. The data was recorded at 250Hz. Additionally, a band-pass and notch filter were respectively applied between 0.5 and 30hz, and 58hz and 62hz with the proprietary g.NEEDaccess python client from G.tec^25^. All the configuration parameters for the device were chosen based on previous studies with the G.Nautilus^25,26^. The channel AFZ is the device’s ground, and the reference is the right earlobe. The preprocessing steps were minimized to allow easy translation to real-time scenarios.

Eye movements were recorded with a Tobii Pro Glasses 2.0 (Tobii Technology AB, Danderyd, Sweden). This device has a pair of inner cameras that precisely track the eye movements and the user’s pupil diameter. This sensor provided 2D and 3D gaze positions and the pupil diameter of both eyes at a sampling rate of 60Hz. No further preprocessing steps were done to the features provided by the sensor. Synchronization and recording of the eye tracker and EEG signals were achieved with the LabStreamingLayer (LSL) software^27^.

#### EEG frequency features

**Figure 3:**
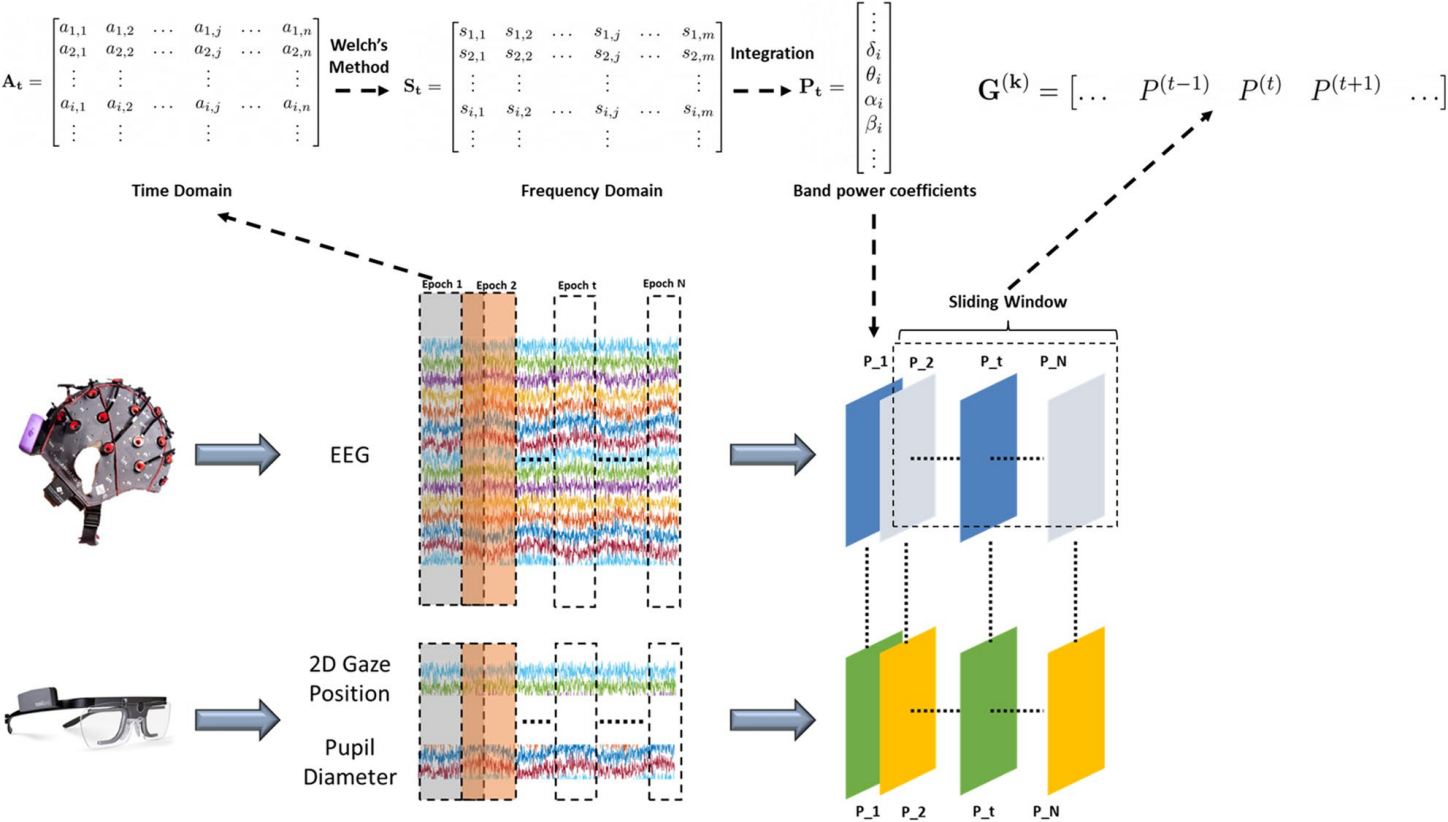
Diagram showing how the EEG and eye tracker signals are synchronized for the cognitive load detection system.

Previous studies^28–30^ have shown that EEG spectral features such as the band power coefficients are correlated to the cognitive workload. Therefore, this representation was used to train our recurrent model. Figure 3 illustrates the pipeline to transform EEG signals into a temporal sequence of band power feature vectors.

First, EEG signals from each recording were split into non-overlapping epochs of 5 s. Each epoch was represented as a matrix $A^{(t)}$ with 32 rows, each one corresponding to a channel of the EEG headset, and *n* columns, corresponding to the number of data points in each epoch. Thus, the entry $a_{i,j}$ represents the $j{th}$ raw sample of the $i{th}$ channel. Then, the power spectral density (PSD) was calculated for every row in matrix $A^{(t)}$ using Welch’s method with a sliding window of 4 s. The resulting PSDs were concatenated into a new matrix $S^{(t)}$.

Finally, band power coefficients were calculated from the channels’ PSDs. These are summary statistics that indicate the energy contribution of specific frequency bands. Following the brain theory of neural oscillations^18^ coefficients from four frequency ranges are extracted: delta band (0.5–4), theta band (4–8Hz), alpha band (8–12Hz), and the gamma band (12–30Hz). Additionally, the coefficients were normalized to the [0–1] range by dividing the band values by the total energy of the signal. This process resulted in the normalized feature vector $P^{(t)}$ of 128 elements (32 channels $\times$ 4 coefficients).

For classification with the recurrent model, a sliding window was used to group the feature vectors of consecutive epochs into a matrix $G^{(k)}$ of dimensions $(128\times L)$, where *L* is the size of the temporal sequence. For the EEG and eye tracker workload classification model a single vector of EEG features, $H^{(k)}$, of size $(128\times 1)$ is calculated by averaging all the feature vectors from the temporal sequence $G^{(k)}$. This vector is further reduced by averaging the bandpower coefficients across all the channels into a feature vector that only contained 4 coefficients. This operation was performed to obtain a similar number of EEG and eye tracker features. The goal of the models was to classify the matrix $G^{(k)}$ or $H^{(k)}$ as either *low-cognitive* (LCL) or *high-cognitive* (HCL).

#### EEG scalp topographical maps

To visually inspect the EEG spectral features, scalp topographical plots for each band power coefficient (delta, theta, alpha, beta) were calculated. These plots showed changes in the spectral coefficients’ spatial distribution between the *(HCL)* and *(LCL)* conditions. In this regard, red regions indicated channels whose mean spectral coefficients had higher magnitude in HCL than in LCL condition, blue regions indicated channels whose mean spectral coefficients had a lower magnitude in HLC than in LCL, and green regions indicated channels where there were no differences between the two conditions. The mean spectral coefficients for each channel were calculated by averaging the extracted features of each trial of each participant. See *Bashivan et al.*^16^ for more details on how to obtain a 2D activity map from the 3D locations of the EEG electrodes.

#### Eye tracker features

The eye tracker provided pupil diameter and 2D gaze position at a sampling rate of 60 Hz. First, the raw signals were divided into 15 s epochs to extract 5 features related to workload: (1) average pupil diameter ${\hat{PD}}$, (2) number of fixations (*NF*), (3) average fixation time(${\hat{FT}}$), (4) scan path length (*SSP*), and (5) nearest neighbor index (*NNI*). The following metrics were selected since they have been previously related to mental workload demands^31^. Fifteen s was the minimum epoch size to calculate the eye tracker features based on fixations. First, the average left eye pupil diameter ${\hat{PD}}$ was calculated for each window using the following equation

1 $$\begin{aligned} {\hat{PD}} = \frac{1}{N}\sum _{i=0}^N PD_i \end{aligned}$$

To calculate the remaining features, the 2D gaze was transformed into a sequence of fixations points. A fixation was defined as the period where the gaze is relatively stationary^32^. Each fixation was associated with a corresponding duration in milliseconds. In this experiment, only fixations of at least 85ms were considered. After obtaining the fixations of each window, the total number of fixations (*NF*) and the average fixation time (${\hat{FT}}$) were calculated. Then, the scan path length was computed as the total Euclidean distance between consecutive fixations with the following equation

2 $$\begin{aligned} SSP = \sum _{i=2}^{NF} d(f_{i-1},f_i) \end{aligned}$$

where d is the Euclidean distance operator and $f_i$ is the $i\text{th}$ fixation. Last, the nearest neighbor index was calculated as the ratio of the nearest neighbor distance of fixations *d*(*NN*) and the average distance of a randomly distributed set of fixations *d*(*ran*). The nearest neighbor distance, *d*(*NN*), was calculated by applying equation 3 to the fixations set^31^. *d*(*ran*) was calculated by applying 3 to a randomly generated set of fixations.

3 $$\begin{aligned} d(NN) = \sum _{i=1}^{NF} \left[ \frac{min(d_{ij})}{N}\right] , 1 < j < NF \end{aligned}$$

#### Recurrent architecture for EEG classification (Dataset 1)

Classification of spectral feature vectors from the EEG was achieved with a compact architecture based on gated recurrent neural networks. This model was selected because of its capability to model the long-term dependencies. The architecture consisted of two stages and was implemented in the Keras framework^33^. The first stage consisted of a fully connected layer that reduced the dimensionality of the input feature vector $P^{(t)}$. In the second stage, cognitive feature vectors from multiple time steps were combined with two bidirectional Long Short-Term Memory (LSTM) layers^34^ to predict the final workload.

To train the described model, a binary cross-entropy loss function shown in equation 4 was used where $L_i$ is the label for the $i{th}$ input sequence, *N* is the number of training samples, and $y_i^{(T)}$ is the output of the last LSTM cell. To optimize the model architecture, a grid search cross-validation of the following hyperparameters was conducted: (1) number of LSTM layers, (2) connectivity of the recurrent layers, i.e., unidirectional or bidirectional, and (3) dropout rates. The dropout rate adopted was from 0.5 to 0.45 with step size 1 and the number of LSTM layers from 1 to 3 with a step size of 1.

4 $$\begin{aligned} Loss = -\frac{1}{N}\sum _{i=0}^N L_i \cdot log\left( y_i^{(T)}\right) + (1-L_i) \cdot log\left( 1-y_i^{(T)}\right) \end{aligned}$$

#### Feed forward model for multi-sensor classification (dataset 2)

To classify signals from multiple sensors, a feed-forward neural network was used. The network used only a combination of fully connected layers with Relu activation functions, dropout, and batch normalization layers. To train the described model, the binary cross-entropy function (see equation 4) was used and the Adam^35^ optimization algorithm. Models were trained for 100 epochs using a batch size of 10 samples.

To optimize the model architecture, a grid search cross-validation of the following hyper-parameters was conducted: dropout rate and the number of hidden layers. The dropout rate from 0.5 to 0.45 was tested with step size one and the number of hidden layers from 4 to 8 with a step size of 1.To train, optimize and evaluate the proposed models, data from each user was split using a ratio of 60-20-20. The first 60% of the data was used to train the architectures, the next 20% to optimize the architectures and the last 20% to test the algorithms.

### Robotic module

#### Robot description and user teleoperation

Our autonomous robotic module was deployed in a Da Vinci Research Kit Robot (DVRK)^15^. This robot is composed of 4 teleoperable robotic manipulators: (1) three Patient Side Manipulators (PSMs), (2) an Endoscopic Camera Manipulator (ECM). The surgical console of this system contains an immersive display that provides a 3D view of the surgical field and master tool manipulators that allow controlling the robotic manipulators on the patient side. For this work, only two out of the three teleoperable PSMs were directly controlled by the user while the third one was controlled by the autonomous algorithms. This scheme allowed us to evaluate both the performance of the autonomous suction assistant and the interaction between the user and the autonomy.

#### Robot autonomy

**Figure 4:**
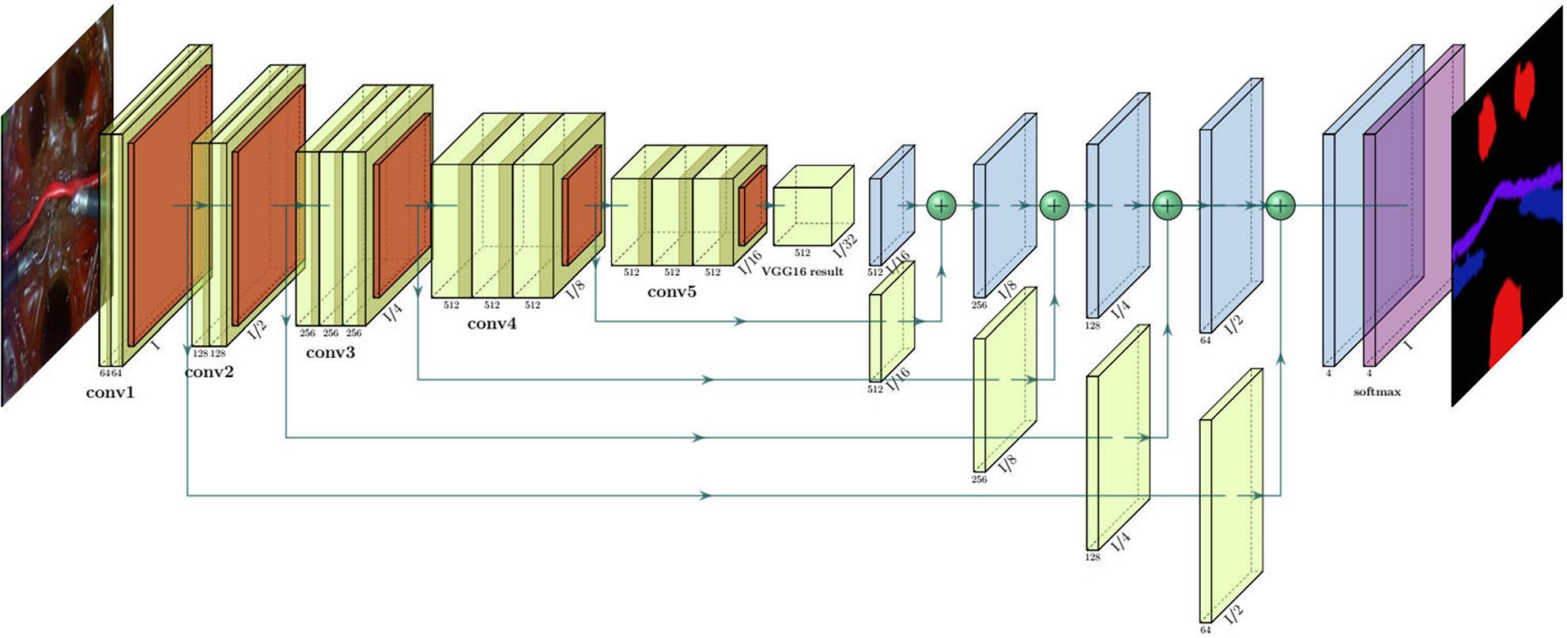
Proposed fully convolutional network with a VGG-16 backbone. The architecture uses the following color coding: (1) green blocks represent convolutional layers, (2) orange blocks represent max-pooling layers, (3) blue blocks represent upsampling layers and (4) purple layers represent softmax layers.

Our autonomous robotic assistant is composed of two modules: (1) a computer vision system to automatically segmented blood accumulations in the surgical field and (2) a path planner module that transformed the pixel locations of the blood accumulations into spatial coordinates used to create the suction trajectory. The automatic segmentation of blood in the surgical field was performed with a fully convolutional network based on the VGG-16 architecture (See Fig. 4). This network allowed to produce real-time segmentation maps of the blood accumulations, that were used to guide the robotic assistant. For training purposes, we collected a dataset of 180 images extracted from endoscopic videos while the robot was teleoperated in our surgical simulator. Additionally, two techniques were utilized to prevent overfitting of the models in our dataset. First, the backbone’s weights of our FCN model were initialized with the weights of a VGG-16 pre-trained in the ImageNet dataset. Second, our dataset was augmented utilizing spatial and color-space data augmentations techniques. A more thorough description of the calibration and training of our robotic module can be found in previous work^36^.

On deployment, the robot automatically segmented the endoscopic images with the FCN model and calculated the centroids and areas of all the detected blood blobs. Then, the pixel coordinates to the blob with the biggest area are calculated. These target pixel coordinates are then transformed using a homography into spatial coordinates for the surgical robot. This homography was calculated during a hand-eye calibration procedure performed at the beginning of the experiment. Last, a straight-line trajectory from the current position to the calculated target position was calculated and executed by the robot. To avoid collisions between the suction arm and teleoperated arms, we opted to provide an AR cue to the operator to let him know the current target of the robot, so he can coordinate his movements to avoid collisions. This is a more effective approach given that the surgeon hands are constantly moving (See supplementary video). The centroids of the accumulations were selected as the target location to maximize the amount of blood that the robot could draw in a single retraction motion.

### Experiment 1: evaluation of the neurotechnology based real-time workload assessment system

To build the dataset of physiological signals, two user studies were conducted at Purdue University. Both studies were approved by the Purdue University Institutional Review Board (IRB) protocol 1906022354. All the experiments were performed in accordance with Purdue’s IRB guidelines and regulations. Additionally, informed consent was obtained from all the participants prior to the experiment. For dataset 1, a user study with 8 participants was conducted. Recruited participants were asked to perform the peg transfer task, a crucial part of the fundamentals of robotic surgery^11^.

This peg-transfer task was designed to have two difficulty levels to elicit different states of cognitive load in the user. The easy task or less cognitive demanding task was the peg-transfer performed with the normal teleoperation mode of the robot. In this condition, the surgeon’s hand and the robotic gripper would move in the same direction, e.g., when the surgeon’s hand moves to the right, the robotic gripper moves to the right as well. The difficult task introduced a motion reversal effect in the teleoperation of the robot. In this condition, the robot’s tooltip moved in the opposite direction from the user’s hand movement, e.g., when the surgeon’s hand moves to the right, the robotic tooltip moves to the left (opposite direction). This inversion effect emulated the fulcrum motion effect seen in traditional laparoscopic surgery^37^. This task was inspired by the studies showing how mirroring the hand movements of a previously learned task resulted in significant differences in the EEG spectral content^38^.

During each session, the user performed six trials of the peg transfer task, three times in the easy level and three times in the difficult level, each one for 5 minutes, accounting for the 30 minutes of EEG data per session. Finally, the difficulty level was used to partition the EEG trials into two categorical labels: low cognitive load and high cognitive load. Each of the users came for 4 sessions of data collection. Each session happened on a different day.

To build dataset 2, a new user was conducted with 10 students at Purdue University. In this study, participants were required to teleoperate the DVRK robot to perform a needle pass task^39^ in our bleeding simulator while wearing the EEG and eye tracker sensors. To elicit different workload demands, the task was designed to have two different levels of difficulty. In the low workload task, users performed the needle pass exercise with no bleeding events. In the high workload task, users completed the task as the cavity filled up with blood. Ground truth labels for our dataset were assigned depending on the difficulty of the task. Users performed 10, 3 minutes trials, alternating between the difficult task and the easy task. This protocol accounted for 30 minutes per session. Each user only performed 1 data collection session.

### Experiment 2: integration of robotic autonomous assistant and cognitive prediction module

To evaluate the integration between the cognitive workload sensing module and autonomous suction assistant, a user study was conducted with 8 students. This study was approved by the Purdue University’s Review Board under the protocol IRB-2021-22. As in experiment 1, experiments were performed following Purdue’s IRB guidelines and regulations, and consent was obtained from all the participants prior to the experiment. For this study, the users were asked to come for two sessions occurring on subsequent days. In the first session, the users were allowed to practice until they become proficient at teleoperating the robotic platform to perform the surgical exercise. For this experiment, the same surgical tasks as in dataset 2 were used. On the second day, we asked the users to wear the EEG and eye tracker sensors before starting the experiments. After that, they performed the assigned tasks under two different modalities: manually-teleoperated suction action(*MS*) and autonomous-suction action by the robotic assistant (*AS*). In *MS*, the users manually teleoperated the suction tool to facilitate their task. In *AS*, the autonomous robotic assistant controlled the suction tool. The underlying hypothesis is that the autonomous suction action (*AS*) will lead to better performance and lower mental demands than the manual teleoperation (*MS*).

In *AS* modality, the user was instructed to teleoperated PSM1 and PSM2 while the autonomous algorithms controlled the PSM3. In *MS*, the user had to swap the teleoperation between the main instrument arms and the suction tool by pressing the clutch pedal in the console. After completing each task, the user was asked to answer a NASA-TLX (National Aeronautics and Space Administration Task Load Index) questionnaire. During the experiment, kinematic data and the endoscopic video were recorded from the robot to assess the user’s performance in each modality. Each subject completed the surgical tasks using both modalities (MS and AS). Thus, a paired t-test was used to evaluate the differences in performance and workload metrics. P-values below 0.05 were assumed to be statistically significant in this study.

### Evaluation metrics

To evaluate the autonomous system’s performance, a combination of objective performance and workload metrics were used. The workload metrics included a NASA-TLX score and a workload prediction score. The objective performance metrics included kinematic, video information, and collaboration fluency metrics. These metrics are time, collaboration fluency, motion, system events, and blood metrics. Collaboration fluency refers to the capability of a human and robot to work as a team proficiently towards a common goal^40^. In this regard, we used the following metrics. (1) Human idle time, measured by the idle time associated with the use of the main instrument arms (PSM1 and PSM2). (2) Robot idle time, measured as the percentage of time that the autonomous assistant (PSM3) was idle. (3) Concurrent activity, measured as the percentage of time that both the autonomy and human were simultaneously active. Low human and robot idle time and high concurrent activity time are indicators of effective human–robot collaboration. Blood metrics were calculated as the average number of pixels corresponding to blood regions, which is a proxy for the volume of blood accumulated, assuming a constant depth. This calculation was accomplished by running our semantic segmentation algorithm on the recorded videos of each user.

## Supplementary Information

Supplementary Information 1.

Supplementary Information 2.

## Data Availability

The datasets generated during and/or analyzed during the current study are available from the corresponding author on reasonable request.
