# Supplementary material for: A neurotechnological aid for semi-autonomous suction in robotic-assisted surgery
Source: Sci Rep. 2022 Mar 16;12:4504. doi: 10.1038/s41598-022-08063-w (PMC8927583; doi:10.1038/s41598-022-08063-w)
Supplement: Supplementary file 2 — Supplementary Information 2. [file 41598_2022_8063_MOESM2_ESM.docx]

**Video title**

Supplementary video 1.

**Video legend**

Supplementary video 1 shows the second experiment of the paper. In this experiment, the user and autonomous system collaborated during periods of high cognitive workload. Additionally, the video shows the semantic segmentation maps driving the autonomous system in real-time.
